# Effectiveness of eLearning and blended modes of delivery of Mental Health First Aid training in the workplace: randomised controlled trial

**DOI:** 10.1186/s12888-018-1888-3

**Published:** 2018-09-26

**Authors:** Nicola J. Reavley, Amy J. Morgan, Julie-Anne Fischer, Betty Kitchener, Nataly Bovopoulos, Anthony F. Jorm

**Affiliations:** 1 Centre for Mental Health, Melbourne School of Population and Global Health, University of Melbourne, 207 Bouverie Street, Carlton, VIC 3010 Australia; 2 Mental Health First Aid Australia, 369 Royal Parade, Parkville, VIC 3052 Australia

## Abstract

**Background:**

The aim of the WorkplaceAid study was to compare the effects of eLearning or blended (eLearning plus face-to-face course delivery) Mental Health First Aid (MHFA) courses on public servants’ knowledge, stigmatising attitudes, confidence in providing support and intentions to provide support to a person with depression or post-traumatic stress disorder (PTSD).

**Methods:**

A randomized controlled trial was carried out with 608 Australian public servants. Participants were randomly assigned to complete an eLearning MHFA course, a blended MHFA course or Red Cross eLearning Provide First Aid (PFA) (the control). The effects of the interventions were evaluated using online questionnaires pre- and post-training. The questionnaires centred around vignettes describing a person meeting the criteria for depression or PTSD. Primary outcomes were mental health first aid knowledge and desire for social distance. Secondary outcomes were recognition of mental health problems, beliefs about treatment, helping intentions and confidence and personal stigma. Feedback on the usefulness of the courses was also collected.

**Results:**

Both the eLearning MHFA and blended MHFA courses had positive effects compared to PFA eLearning on mental health first aid knowledge, desire for social distance, beliefs about professional treatments, intentions and confidence in helping a person and personal stigma towards a person with depression or PTSD. There were very small non-significant differences between the eLearning MHFA and blended MHFA courses on these outcome measures. However, users were more likely to highly rate the blended MHFA course in terms of usefulness, amount learned and intentions to recommend the course to others.

**Conclusions:**

The blended MHFA course was only minimally more effective than eLearning MHFA in improving knowledge and attitudes. However, course satisfaction ratings were higher from participants in the blended MHFA course, potentially leading to greater benefits in the future. Longer-term follow-up is needed to explore this.

**Trial registration:**

ACTRN12614000623695 registered on 13/06/2015 (prospectively registered).

## Introduction

Because of the high prevalence of mental disorders, members of the public are very likely to have contact with people with mental health problems (which includes those with diagnosed disorders or subclinical symptoms) and can play a valuable role in providing support [1, 2]. It can therefore be argued that the public need knowledge and skills to provide help to people with mental health problems. This help, also known as ‘mental health first aid’, can be defined as the help provided to a person developing a mental health problem, experiencing a worsening of a mental health problems or in a mental health crisis (e.g. at immediate risk of suicide) [3]. The first aid is given until appropriate professional treatment is received or until the crisis resolves. The Mental Health First Aid (MHFA) training course teaching these skills was developed in Australia in 2000 [4]. It involves 12 hours of face-to-face instruction and gives an overview of the most common and disabling mental health problems, introduces a five-component action plan and then applies these actions to help people with problems of depression, anxiety disorders, psychosis and substance use disorder as well as crisis situations including suicidal behaviours, panic attacks, traumatic events, aggressive behaviour, and drug overdose [5].

Since its inception, MHFA training has expanded rapidly; there are now 1500 accredited MHFA instructors in Australia who have trained over 500,000 adults. It operates in over 20 countries and 2 million people have been trained globally [6]. Evaluation studies were first carried out in Australia and have now been undertaken in other countries. A recent meta-analysis conducted by Morgan et al. [7] included 18 randomised controlled trials that evaluated the effectiveness of MHFA in a range of settings. MHFA training led to improved mental health first aid knowledge with effects persisting up to 12 months after training. There were also moderate improvements in recognition of mental disorders and beliefs about effective treatments and and small reductions in stigmatising attitudes (ds 0.08–0.15). Perceived confidence in helping a person with a mental health problem and intentions to provide first aid were also seen post-training and persisted for up to 6 months later. There were small improvements in the amount of help provided to a person with a mental health problem at follow-up (d = 0.23) although impact of MHFA on the quality of help offered was not clear.

Eight of the studies included in the meta-analysis mentioned above were conducted in workplace settings, which is increasingly seen as an important setting for mental health education and training, not only to address mental health problems caused by work, but also to address mental health problems that arise as a result of other factors but that may become apparent in the workplace or be exacerbated by poor working conditions [8, 9]. Growing evidence suggests that workplace interventions may produce improvements in mental health literacy [10] and depression and anxiety symptoms [9, 11] as well as reductions in stigma [10].

In the context of the growing popularity of eLearning, an electronic version of MHFA was developed and evaluated in a randomised controlled trial (RCT) conducted in 2009 involving 262 members of the public, who were randomly assigned to complete the eLearning course, read a printed MHFA manual or be in a wait-list control group [12]. Participants were sent weekly emails for four weeks to prompt them to continue participating. The impact of the interventions was evaluated using online questionnaires administered at baseline, post-training and at 6-months follow-up. Both eLearning and the printed manual were superior to wait list in increasing knowledge, reducing stigmatising attitudes and increasing confidence. eLearning showed greater effects than wait-list or manual in reducing stigmatising attitudes to a person with schizophrenia and also in improving first-aid actions taken.

As with other intervention settings, recent years have seen an increase in online interventions in the workplace. eLearning may be particularly appropriate to workplace settings, where it is not always easy to roster staff to attend training simultaneously, particularly for longer courses such as face-to-face MHFA training which is 12 hours long and requires four sessions of three hours each. Other groups of people for whom attendance is difficult, are those living in remote areas or who are distant from where courses are offered, shift workers, and people who have family commitments which make it difficult to attend for this length of time. However, one of the disadvantages of the eLearning format is that it lacks the group interaction, discussion and questions that are part of a face-to-face course.

One approach to training that aims to maximise the convenience of eLearning with the interactivity of face-to-face learning, is that of blended learning. Common in the field of health professional education, blended learning has been shown to be more effective or at least as effective as non-blended instruction for knowledge acquisition [13]. However, authors of a recent meta-analysis that included 56 studies found large heterogeneity across studies and noted that their conclusions should be treated with caution [13]. Another meta-analysis of eLearning trials covered 96 studies of 168 training courses [14] and focused on comparisons between face-to-face instruction and blended learning or eLearning. Results showed that, for declarative knowledge (memory of facts and principles taught), effects were larger for blended learning (Cohen’s d = 0.35 (95% CI 0.29–0.39)) than eLearning only (d = 0.15 (95% CI 0.11–0.19)). The findings were similar for procedural knowledge (information about how to perform a task or action): d = 0.53 (95% CI 0.34–0.70) vs d = − 0.07 (95% CI -0.20-0.06). This meta-analysis also looked at learner satisfaction with the mode of instruction and found a very small effect in favour of online compared to blended learning. However, the studies included in these reviews focussed specifically on its effectiveness with adults acquiring knowledge directly relevant to their current or future employment. There has been very little research into the effectiveness of mode of instruction for interventions targeted to the general public where the content does not relate to employment. Furthermore, a review of anti-stigma interventions found no difference in effects between studies with internet or non-internet delivery of content, but head-to-head comparisons of delivery mode are rare [15]. No prior studies have compared the effectiveness of MHFA training when delivered in an eLearning versus blended mode.

The principal aim of this study was to compare the effects of eLearning or blended (eLearning plus face-to-face course delivery) MHFA training on knowledge, stigmatising attitudes, confidence in providing support and intentions to provide support to a person with depression or PTSD. Participants were members of the public service in Victoria and the Australian Capital Territory (ACT), Australia.

Study hypotheses were as follows:

1. Compared to eLearning Provide First Aid (PFA) training (the control), MHFA training, whether delivered in eLearning or blended modes, will result in higher mental health first aid knowledge, lower stigmatizing attitudes, and greater confidence in providing support to a person developing a mental health problem or in a mental health crisis.
2. Compared to eLearning MHFA training, blended MHFA training will result in higher mental health first aid knowledge, lower stigmatizing attitudes, and greater confidence in providing support to a person developing a mental health problem or in a mental health crisis.
3. Compared to eLearning MHFA training, blended MHFA training will be rated higher on satisfaction than eLearning MHFA training.

## Methods

### Study design

The WorkplaceAid study was a parallel group RCT with participants randomized to eLearning MHFA, blended MHFA or PFA eLearning in a 1:1:1 ratio. The trial was registered with the Australian and New Zealand Clinical Trials Registry (ACTRN12614000623695, registered on 13 June 2014). PFA eLearning was chosen as a control intervention as it deals with health problems that may occur in the workplace, uses a similar mode of administration to the eLearning MHFA training and involves a similar time commitment.

### Participants

Australian public servants who were aged 18 and over and did not hold a current certificate in either MHFA or PFA were eligible for the study. Initially, state government employees in the state of Victoria, Australia, were eligible to participate. In 2016, recruitment was opened to Commonwealth government employees in the ACT in order to counteract a slow-down in recruitment in the Victorian public service after the 2014 state election and subsequent department restructure. Participants were informed about the study through flyers put up in offices, articles in newsfeed items on the staff intranet and at staff wellbeing events. Potential participants were asked to register online by going to a trial website, which contained a Participant Information Sheet, an ‘I accept’ checkbox and a link to the baseline SurveyMonkey questionnaire. After completion of this questionnaire, participants were randomized to one of the three groups listed above and given instructions on how to access the relevant course. Participants were advised that they could complete the training during work hours, with their manager’s approval, avoiding busy times at work.

### Randomization

Randomization was carried out by a random integer generator programmed on the trial website to give values of 1 = eLearning MHFA; 2 = Blended MHFA and 3 = PFA eLearning (see http://php.net/manual/en/function.rand.php). Allocation was concealed as randomization was computer generated. Blinding was not possible due to the nature of the interventions.

### Interventions

#### eLearning MHFA

This intervention was a 6-hour eLearning MHFA course accessed via the MHFA Australia online portal. The course teaches a 5-component action plan for responding to developing mental health problems such as depression, anxiety problems, psychosis and substance use problems, and crises including suicidal thoughts and behaviours, non-suicidal self-injury, panic attacks, traumatic events, severe psychotic states, severe effects from alcohol misuse, severe effects from drug misuse, and aggressive behaviours. The eLearning MHFA course comprised 5 modules which needed to be completed in sequence, with a quiz at the end of each: Introduction to mental health; Depression; Anxiety Problems; Psychosis and Substance Use Problems. Each module included interactive content on case studies (click on a picture or table to answer). The course also includes audio and video content depicting stories of lived experience and demonstrating how to provide mental health first aid, following by questions or other activities.

The online content was tailored to incorporate information on resources and help-seeking pathways of specific relevance to the relevant public service (e.g. Employee Assistance Program (EAP) providers). An accompanying hard copy MHFA manual (which was regularly referred to in the eLearning course) was posted to participants when they registered for the course [5]. Participants received weekly automated emails for 6 weeks to pace them through the material. Monthly reports were extracted to monitor course progress. On course completion, an automatic email was sent to the trial manager to flag completion and another was sent to the participant containing a link to the post-course questionnaire.

#### Blended MHFA

This intervention included the 6-hour eLearning course described above plus a 4-hour face-to-face session, which reviewed the contents of the online course through quizzes and discussion. It also included case studies and role plays to give participants more experience in applying the MHFA Action Plan in different situations and settings. Group training was completed within 3 months of online course completion. In Melbourne and Ballarat, Victoria, two MHFA Instructors ran 16 courses individually, while one Instructor delivered 9 courses in Canberra. Courses were held in locations close to participants’ workplaces.

#### Provide first aid

This intervention was a 4-hour eLearning PFA course delivered via the Australian Red Cross online portal. The course teaches the fundamental principles, knowledge and skills to provide emergency care for injuries and illnesses in the home or the workplace. Participants received weekly emails for 6 weeks to pace them through the material. Reports from Red Cross were obtained to monitor course progress and flag course completers.

Following online completion, participants in each course were awarded an online ‘Certificate of Completion’. For the MHFA courses, participants could undertake an exam (within 3 months of course completion) to become an ‘Accredited Mental Health First Aider’, valid for 3 years. Staff who completed the eLearning PFA course were offered an option to attend a 1-day assessment (in their own time) to obtain a ‘Statement of Attainment’ for the Health Training Package, HLTAID003 – Provide First Aid, also valid for 3 years.

### Outcomes

Data were collected at baseline, post training (participants were given 2 months to complete the assessment after they had completed the course), one-year follow-up and two-year follow-up. Only outcomes assessed at baseline and post training are reported here. Data from the one and two- year follow-up questionnaires will be reported in a future publication as data collection is ongoing. Participants were invited to complete an online questionnaire via SurveyMonkey after they had completed the eLearning course, or had attended the face-to-face course. The median length of time between baseline and post training assessment was 85 days.

Initial questions covered sociodemographic information (age, gender, email, daytime telephone number, employment time fraction, marital status, postcode, country of birth, language spoken at home, level of education, Aboriginal and Torres Strait Islander status and whether the respondents managed staff). Subsequent questions centred around a vignette of a person ‘John’ meeting the DSM criteria for major depression with suicidal thoughts and ‘Paula’ meeting the DSM criteria for PTSD (previously validated vignettes of two mental disorders which have high prevalence rates and are therefore relatively likely to be encountered in the workplace) [2, 16].

#### Primary outcomes

These were as follows:

##### Mental health first aid knowledge

This was measured using a 16-item true/false quiz based on the content of the Mental Health First Aid Manual [5]. Respondents were presented with the statements given in Table 1 and asked whether they agreed, disagreed or did not know. The number of correct responses was converted into a percentage. A Pearson correlation coefficient was used to assess test-retest reliability in the control group, which was 0.61 (95% CI 0.44–0.73).

**Table 1:** Mental health first aid knowledge

1) Half of all people who experience a mental illness have their first episode by age 18	
2) Depressive disorders are the most prevalent mental illness in the Australian population	
3) If a person who is depressed does not want to seek professional help, it is important to force them to if you can	
4) Exercise can help relieve depression	
5) Recovery from anxiety disorders requires facing situations which are anxiety provoking	
6) Antidepressant medications can be an effective treatment for most anxiety disorders	
7) When interacting with a person with psychosis, it is best not to offer them choices of how you can help them because it could add to their confusion	
8) A person with a psychotic illness is less likely to relapse if they have a good relationship with their family	
9) A good way to help a person with a drug or alcohol problem is to let them know that you strongly disapprove of their substance use	
10) People with mental illnesses are much more likely to be smokers	
11) It is not a good idea to ask someone if they are feeling suicidal in case you put the idea in their head	
12) It is best to get someone having a panic attack to breathe into a paper bag	
13) If someone has a traumatic experience, it is best to make them talk about it as soon as possible	
14) It is best not to try to reason with a person having delusions	
15) If a person is intoxicated with alcohol, it is not possible to make them sober up more quickly by giving them strong coffee, a cold shower or taking them for a walk	
16) If a person becomes unconscious after taking drugs, it is best to lie them on their side rather than on their back	

##### Desire for social distance

This was measured by the Social Distance Scale [17] in relation to the vignettes described above. This scale is widely used, and many studies have shown that it has a single factor structure [18, 19]. Respondents were asked: Would you be happy to: 1) To move next door to John/Paula? 2) To spend an evening socializing with John/Paula? 3) To make friends with John/Paula? 4) To work closely with John/Paula on a project at work? 5) To have John/Paula marry into your family? Responses were scored as follows: 1 = Yes definitely (low social distance) to 4 = Definitely not (high social distance). The internal consistency of the scales in this sample (omega total) was 0.88 for depression and 0.91 for PTSD. Scores were dichotomised at the median of pre- and post-scores.

#### Secondary outcomes

##### Recognition of mental health problems

In order to assess recognition of the problems in the vignettes, participants were asked an open-ended question, “What, if anything do you think is wrong with John/Paula?” For the depression vignette, participants were assessed as having correctly recognised the problem if they mentioned ‘depressed’ or ‘depression’ in their response. For the PTSD vignette, responses with any mention of PTSD, post-traumatic stress, or post-traumatic stress disorder were assessed as correct. Pearson correlation coefficients were used to assess test-retest reliability in the control group, which were 0.56 (95% CI 0.39–0.70) for depression and 0.65 (95% CI 0.50–0.77) for PTSD.

##### Beliefs about treatment

These were assessed using a 16-item scale based on the 2011 National Survey of Mental Health Literacy and Stigma [20] and a consensus between Australian clinical psychologists, psychiatrists, and GPs established by a national survey [21]. Respondents were presented with sources of potential help for depression and PTSD. For the depression vignette, participants scored 1 point for ‘Helpful’ responses to each of the following: a typical family GP or doctor; a psychiatrist; a psychologist; becoming more physically active; reading about people with similar problems and how they have dealt with them; psychotherapy; cognitive behaviour therapy; cutting out alcohol altogether; and antidepressants. They also scored 1 point for rating ‘dealing with the problem alone’ as harmful. For the PTSD vignette, participants scored 1 point for ‘Helpful’ responses to each of the following: a typical family GP or doctor; a psychiatrist; a psychologist; becoming more physically active; reading about people with similar problems and how they have dealt with them; courses on relaxation, stress management, meditation or yoga; psychotherapy; cognitive behaviour therapy; and receiving information about his problem from a health educator. Depression treatment beliefs ranged from 0 to 10 and PTSD treatment beliefs from 0 to 9. Pearson correlation coefficients were used to assess test-retest reliability in the control group, which were 0.58 (95% CI 0.40–0.71) for depression and 0.57 (95% CI 0.40–0.70) for PTSD.

##### Helping intentions and confidence

Intentions to provide help to the person in the vignette were assessed by asking respondents: If John/Paula was a co-worker, I would help him/her. This was scored using a 7-point scale ranging from 1 = Strongly Disagree to 7 = Strongly Agree. Responses were coded into two categories: strongly agree/agree vs other. Spearman correlation coefficients were used to assess test-retest reliability in the control group, which were 0.45 (0.25–0.61) for depression and 0.43 (0.23–0.60) for PTSD. This was followed with, “Describe all the things you would do to help John/Paula.” (open-ended response). Scoring was based on the MHFA Action Plan [22] with quality of intended support ranging from 0 to 12. Open-ended responses were scored blinded to allocation or occasion. A random sample of 50 responses were double-coded for each vignette, and inter-rater reliability (ICC) was 0.88 for depression and 0.94 for PTSD. Pearson correlation coefficients were used to assess test-retest reliability in the control group, which were 0.18 (95% CI -0.04, 0.39) for depression and 0.42 (95% CI 0.21–0.59) for PTSD.

Confidence in providing help to someone at work with depression and PTSD was assessed by asking participants, “How confident do you feel in helping someone at work with a problem like John/Paula?” Confidence was rated using a 5- point scale used in previous studies, 1 = Not at all to 5 = Extremely [4]. Pearson correlation coefficients were used to assess test-retest reliability in the control group, which were 0.64 (0.49–0.76) for depression and 0.50 (0.32–0.65) for PTSD.

##### Personal stigma

The Personal Stigma Scale was used to measure participants’ stigmatising attitudes to the person described in the vignette [23]. Exploratory Structural Equation Modelling has shown this scale to have factors measuring belief that a person with a mental health problem is weak not sick, and belief that they are dangerous or unpredictable [19]. This scale includes the following 9 items: 1) (John/Paula) could snap out of it if they wanted; 2) (John/Paula)‘s problem is a sign of personal weakness; 3) (John/Paula)‘s problem is not a real medical illness; 4) (John/Paula) is dangerous; 5) It is best to avoid (John/Paula)‘s so that you don’t develop this problem yourself; 6) (John/Paula)‘s problem makes him/her unpredictable; 7) You would not tell anyone if you had a problem like (John/Paula)‘s; 8) I would not employ someone if I knew they had a problem like (John/Paula)‘s; 9) I would not vote for a politician if I knew they had suffered a problem like (John/Paula)‘s. Scoring ranged from 1 = Strongly Agree to 5 = Strongly Disagree. The Omega coefficient in this sample for’ Weak not sick’ stigma was 0.83 for depression and 0.82 for PTSD. For Dangerous/unpredictable stigma, the Omega coefficient was 0.70 for depression and 0.72 for PTSD. ‘Weak not sick’ scores showed substantial negative skew so were dichotomised at the median, which was the highest possible score (5) for both depression and PTSD vignettes.

##### Course satisfaction

Course satisfaction was assessed using the following questions: 1) How much of the training did you complete? (1 = none of it, 2 = part of it, 3 = most of it, 4 = all of it); 2) How easy was the material to understand? (1 = very easy, 2 = easy, 3 = neither easy nor difficult, 4 = difficult, 5 = very difficult);

3) How much did you learn from the course? (1 = a great deal, 2 = a fair bit, 3 = not very much, 4 = almost nothing); 4) How useful was the course? (1 = very useful, 2 = useful, 3 = not very useful, 4 = not at all useful); 5) Do you think you will use the training material in the future? (1 = yes, 2 = no, 3 = not sure); 6) Would you recommend the course to others? (1 = yes definitely, 2 = probably, 3 = probably not, 4 = definitely not); 7) What did you like about the training materials? 8) What did you dislike about the training materials? These questions were asked post-course only.

### Sample size estimation

In order to calculate the required sample size, we considered the main hypothesis of interest to be the following: that blended MHFA training would be superior to MHFA eLearning in achieving improvements in MHFA knowledge and reductions in desire for social distance. The sample size required to detect differences between these two modes of training was larger than that required to detect differences between these modes and PFA training. Consequently, we chose a small effect size to evaluate the difference between the two modes of MHFA training, as an effect size smaller than this may not be meaningful in terms of participant outcomes. According to Stata Release 12, 165 participants were required per group. For a repeated measures design, with 1 pre-training measure and 3 post-training measures, using the change method of sample size calculation and assuming a conservative 0.70 correlation between pre- and post-measurements (based on [24]), to detect an effect size of Cohen’s d = 0.20 (or h = 0.20), with a power = 0.80 and an alpha = 0.05, Increasing the sample size by 20% to account for attrition, the total sample size required was estimated to be 594 (198 participants per group).

### Adverse events

In the event that a participant felt distressed during survey completion or while undertaking the training, a list of contacts was included at the end of each online survey. This included phone numbers for Lifeline, Suicideline (Victoria only), SANE, Emergency Services (000) and relevant EAP providers. Lifeline’s Online Crisis Chat link was also included. Participants were encouraged to contact the trial manager to report any adverse events. None were reported.

### Ethics

The study was approved by the University of Melbourne Human Ethics Sub-Committee (Ethics ID 1341345.2).

### Statistical analysis

An intention-to-treat approach was used, with all participants included in the analyses. Data were analysed using mixed-effects models for continuous and binary outcome variables, with group-by-measurement occasion interactions. This method takes into account the hierarchical structure of the data in the analysis of differences between the groups, i.e. the correlation of measurement occasions within participants. It can produce unbiased estimates when a proportion of the participants withdraw before the completion of the study, based on the reasonable assumption that these data are missing at random [25]. As tertiary-educated participants were somewhat more likely to have data at post (OR = 1.39, 95% CI 0.99–1.95, *p* = .057), education was included as a fixed effect in order to help meet the missing at random assumption. For outcome measures with no substantial baseline imbalance, effect sizes (Cohen’s d) were calculated by dividing the difference between the two group means at post-training by their pooled standard deviation. With baseline imbalances, Cohen’s d was calculated by dividing the mean change in each condition by the pooled standard deviation post-training. Analyses were performed in Stata 14 and RStudio and the significance level was set at *p* < .05.

## Results

### Participant flow and numbers analysed

**Fig. 1:**
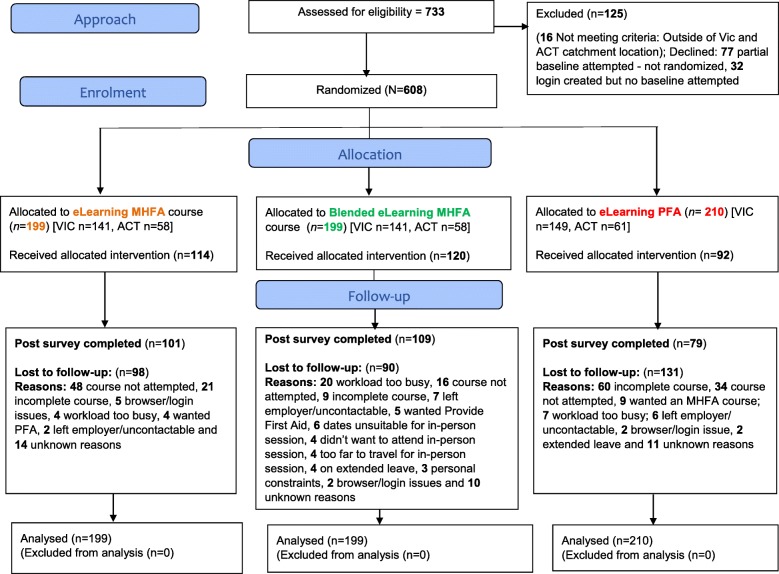
CONSORT flow diagram for the study

The CONSORT flow diagram of the number of participants at each stage of the trial is given in Fig. 1. All the participants included in the analyses completed the first questionnaire and 319 (52.5%) participants did not complete the post-test questionnaire. The sole predictor of having data at post-test was assignment to the PFA group (OR = 0.59, 95% CI 0.39–0.87, *p* = 0.008). The three groups were similar in baseline sociodemographic characteristics, indicating that randomisation resulted in comparable groups (see Table 2).

**Table 2:** Baseline characteristics of participants in intervention and control groups

		Group allocation					
		MHFA eLearning	Blended MHFA	PFA elearning	Total	chi2	*p*
Gender						1.46	0.481
Female	N	148	140	160	448		
	%	74.8	71.1	76.2	74.1		
Male	N	50	57	50	157		
	%	25.3	28.9	23.8	26.0		
Total	N	198	197	210	605		
Only English spoken at home						4.76	0.093
Yes	N	169	180	178	527		
	%	85.4	91.4	84.8	87.1		
Total	N	198	197	210	605		
Tertiary education						1.78	0.410
Yes	N	126	127	146	399		
	%	63.6	64.8	69.5	66.1		
Total	N	198	196	210	604		
Aboriginal or Torres Strait Islander status						0.34	0.842
Yes	N	2	3	2	7		
	%	1.0	1.5	1.0	1.2		
Total	N	198	197	210	605		
Married/Defacto vs Other						2.46	0.292
Yes	N	121	131	143	395		
	%	61.1	66.8	68.1	65.4		
Total	N	198	196	210	604		
Do you manage staff						0.78	0.676
Yes	N	63	65	61	189		
	%	31.8	33.0	29.1	31.2		
Total	N	198	197	210	605		
						F	p
Age						0.3	0.737
	M	40.89	41.71	41.08	41.23		
	SD	11.30	10.93	10.60	10.93		
	N	198	196	210	604		

### Participants’ characteristics

The mean age of participants was 41.2 years (SD =10.9), 74.1% were female, 87.1% spoke English at home, 66.1% were tertiary educated, 1.2% were Aboriginal or Torres Strait Islander, 65.4% were married or in de facto relationships and 31.2% managed staff (See Table 2).

### Program use

In the MHFA eLearning group, 68.8% attempted the online course compared to 73.4% of those in the blended MHFA group and 73.3% of those in the PFA eLearning group. In the MHFA eLearning group, 55.3% completed the course compared to 64.8% of those in the blended MHFA group and 43.3% of those in the PFA eLearning group, the only difference that reached statistical significance (*p* < 0.001). The MHFA course website automatically captured when each module was completed. The mean number of modules completed (among those who attempted the course) was 4.19 (SD = 1.75) for MHFA eLearning and 4.53 (SD = 1.38) for the blended group. Among all participants these means were 2.88 (SD = 2.42) and 3.32 (SD = 2.33) respectively. In the blended group, 59.8% attended face-to-face training and 58.8% attended this and also completed all five modules. The website captured first and last access of the online courses with timestamps. Among participants in the two MHFA groups, 29.2% spent less than one hour on the course, 21.4% spent 1–2 h, 16.3% spent 2–8 h and 33.1% spent more than 8 h.

### Primary outcomes

Baseline and post scores on all outcome measures are presented in Table 3, which also presents the results from the planned contrasts, estimating the mean difference in change over time between groups.

**Table 3:** Observed means (and standard deviations) and estimated mean differences in change over time between intervention and control groups for all outcome measures

Observed scores									Mean change over time		
Outcome	Assessment	eLearning MHFA		blended MHFA		eLearning PFA			MHFA eLearning vs PFA eLearning	Blended MHFA vs PFA eLearning	MHFA blended vs eLearning
		M	SD	M	SD	M	SD		95% CI	95% CI	95% CI
Knowledge											
Knowledge of MHFA (% correct)	baseline	46.9	15.3	44.6	14.6	44.9	15.3	Mean diff	21.31^***^ 16.77–25.90	25.04^***^ 20.51–29.57	3.70 −0.57 – 7.98
	post	71.5	15.0	73.1	12.7	49.4	18.1	Cohen’s d	1.35 1.02–1.67	1.55 1.22–1.89	0.11 − 0.16 - 0.38
Beliefs about treatment - depression	baseline	5.92	2.32	5.79	2.08	5.77	2.28	Mean diff	1.80^***^ 1.19–2.41	2.01^***^ 1.40–2.61	0.20 − 0.36 - 0.77
	post	7.79	1.90	7.76	1.80	5.90	2.54	Cohen’s d	0.86 0.55–1.17	0.87 0.56–1.18	0.15^#^ − 0.12 – 0.43
Beliefs about treatment - PTSD	baseline	4.87	1.96	4.70	1.92	4.78	1.96	Mean diff	1.54^***^ 0.98–2.11	1.59^***^ 1.03–2.15	0.05 −0.48 - 0.58
	post	7.17	1.81	7.08	1.65	5.74	2.48	Cohen’s d	0.67 0.37–0.98	0.66 0.36–0.96	− 0.01^#^ − 0.26 – 0.28
		N	%	N	%	N	%				
Correct recognition of depression	baseline	190	95.5	176	88.4	190	90.5	Odds ratio	1.05 0.17–6.42	11.11^*^ 1.60–77.28	10.57^*^ 1.47–76.22
	post	94	93.1	103	96.3	69	88.5				
Correct recognition of PTSD	baseline	142	71.4	150	75.4	160	76.2	Odds ratio	1.28 0.38–4.32	3.61 0.96–13.57	2.83 0.84–9.55
	post	78	77.2	94	88.7	61	79.2				
Confidence		M	SD	M	SD	M	SD				
Confidence - depression	baseline	2.87	1.06	2.84	1.09	2.88	1.06	Mean diff	0.43^**^ 0.16–0.69	0.56^***^ 0.29–0.82	0.13 −0.12 - 0.38
	post	3.55	0.90	3.63	0.75	3.15	1.06	Cohen’s d	0.41 0.11–0.71	0.53 0.23–0.83	0.09 − 0.19 - 0.36
Confidence - PTSD	baseline	2.88	1.10	2.83	1.09	2.87	1.15	Mean diff	0.58^***^ 0.28–0.87	0.65^***^ 0.36–0.94	0.07 − 0.20 - 0.35
	post	3.74	0.90	3.78	0.81	3.22	1.11	Cohen’s d	0.52 0.22–0.82	0.59 0.29–0.89	0.04 −0.23 - 0.32
MHFA intentions quality		M	SD	M	SD	M	SD				
Intentions - depression	baseline	3.91	1.73	3.62	1.62	3.60	1.61	Mean diff	2.06^***^ 1.45–2.66	3.00^***^ 2.41–3.60	0.95^**^ 0.38–1.52
	post	5.67	2.35	6.35	2.45	3.31	1.55	Cohen’s d	1.16 0.84–1.48	1.43 1.11–1.76	0.28 0.01–0.55
Intentions - PTSD	baseline	3.06	1.45	2.98	1.52	3.03	1.45	Mean diff	2.30^***^ 1.76–2.85	2.47^***^ 1.93–3.01	0.17 −0.35 - 0.68
	post	5.25	2.33	5.40	2.14	2.94	1.37	Cohen’s d	1.17 0.85–1.49	1.33 1.00–1.65	0.07 −0.21 - 0.34
MHFA intentions – Would help		N	%	N	%	N	%				
Depression - Agree or strongly agree	baseline	156	78.4	149	74.9	158	75.2	Odds ratio	3.33^*^ 1.06–10.44	4.26^*^ 1.39–13.07	1.28 0.42–3.94
	post	87	86.1	93	86.9	56	71.8				
PTSD - Agree or strongly agree	baseline	150	75.4	148	74.4	160	76.2	Odds ratio	22.37^***^ 4.82–103.91	7.33^**^ 2.11–25.50	0.33 0.08–1.35
	post	95	94.1	94	88.7	54	70.1				
Social distance - depression											
% scoring at or below the median. Lower scores mean less social distance	baseline	N	%	N	%	N	%				
		141	70.9	137	68.8	148	70.5	Odds ratio	5.30^*^ 1.26–22.37	2.96 0.82–10.63	0.56 0.15–2.09
	post	87	86.1	88	83.0	59	76.6				
Social distance - PTSD											
% scoring at or below the median. Lower scores mean less social distance	baseline	N	%	N	%	N	%				
		95	47.7	102	51.3	108	51.4	Odds ratio	4.99^*^ 1.37–18.25	2.45 0.74–8.13	0.49 0.15–1.59
	post	60	59.4	67	63.2	41	53.3				
Weak not sick – depression											
% scoring at or above the median	baseline	N	%	N	%	N	%				
		112	56.3	115	57.8	108	51.4	Odds ratio	1.53 0.56–4.16	1.64 0.60–4.46	1.07 0.41–2.80
	post	75	74.3	80	75.5	50	64.9				
Weak not sick – PTSD											
% scoring at or above the median	baseline	119	59.8	120	60.3	116	55.2	Odds ratio	1.20 0.42–3.42	1.89 0.63–5.61	1.58 0.56–4.43
	post	76	75.3	87	82.1	55	71.4				
Dangerous/unpredictable - depression	baseline	M	SD	M	SD	M	SD				
		4.02	0.56	4.02	0.55	4.01	0.56	Mean diff	0.28^***^ 0.13–0.43	0.28^***^ 0.13–0.43	0.00 −0.14 - 0.14
	post	4.26	0.54	4.30	0.52	4.02	0.65	Cohen’s d	0.42 0.12–0.72	0.50 0.20–0.80	0.08 − 0.19 -0.36
Dangerous/unpredictable - PTSD	baseline	4.27	0.50	4.25	0.55	4.24	0.53	Mean diff	0.27^***^ 0.13–0.42	0.28^***^ 0.14–0.43	0.01 −0.13 - 0.15
	post	4.46	0.55	4.48	0.47	4.20	0.64	Cohen’s d	0.44 0.14–0.74	0.52 0.22–0.82	0.04 −0.23 - 0.32

* *p* < .05, ** *p* < .01, *** *p* < .001

# d calculated on change, rather than post due to baseline imbalances

There were significantly greater improvements in knowledge in both MHFA eLearning and blended MHFA groups compared to PFA eLearning. These differences were greater than large in size (d = 1.35 and d = 1.55 respectively). The difference between MHFA eLearning and blended MHFA was not significant.

Those in the eLearning MHFA group were significantly more likely to show a reduced desire for social distance from a person with depression or PTSD than those in the PFA eLearning group. The reduction in social distance in the blended group was not significantly greater than the PFA eLearning group. There were no significant differences between the MHFA eLearning and blended courses.

### Secondary outcomes

#### Recognition of mental disorders

For the depression vignette, correct recognition was high at baseline and there was no difference in change in recognition between MHFA eLearning and PFA eLearning. There were significantly greater improvements in the blended MHFA vs PFA eLearning groups and in the blended MHFA vs MHFA eLearning groups. For PTSD, changes were not significantly different between groups.

#### Beliefs about treatment

Beliefs about treatment for depression and PTSD moved closer to those of health professionals in both the MHFA eLearning and blended MHFA groups compared to PFA eLearning. These differences were large in size for depression (d = 0.86 and d = 0.87 respectively) and medium in size for PTSD (d = 0.67 and d = 0.66 respectively). The differences between MHFA eLearning and blended MHFA were not significant.

#### Quality of helping intentions and confidence

There were greater increases in agreement with intentions to help a person with depression and PTSD in both the MHFA eLearning and blended MHFA groups compared to PFA eLearning. The differences between MHFA eLearning and blended MHFA were not significant.

There were greater improvements in quality of helping intentions for depression and PTSD in both the MHFA eLearning and blended MHFA groups compared to PFA eLearning. These differences were large in size for depression (d = 1.16 and d = 1.43 respectively) and PTSD (d = 1.17 and d = 1.33 respectively). There were also significantly greater improvements (of small effect size) in the blended MHFA group than in the eLearning MHFA group for depression (d = 0.28), although not for PTSD.

There were greater improvements in confidence for depression and PTSD in both the MHFA eLearning and blended MHFA groups compared to PFA eLearning. These differences were medium in size for depression (d = 0.41 and d = 0.53 respectively) and PTSD (d = 0.52 and d = 0.59 respectively). The differences between MHFA eLearning and blended MHFA were not significant.

#### Personal stigma

Belief that people with depression or PTSD are ‘weak not sick’ was low at baseline, with no significant differences in improvement between courses. For beliefs in dangerousness/unpredictability, those in the blended and eLearning MHFA groups were significantly more likely to show reduced stigma towards people with depression and PTSD than those in the PFA eLearning group. There were no significant differences between the MHFA eLearning and blended courses.

### Course satisfaction

Over 90% of participants in all groups reported completing all the course and over 75% of participants in all groups reported finding the material very easy or easy to understand. However, it is interesting to note that the self-reported data differs from the objective data captured from the website, which suggested lower completion rates. Differences between groups did not reach statistical significance. When asked how much they had learnt from the course, 61.5% of participants in blended MHFA reported learning a great deal, compared to 36.6% in the eLearning course and 30.4% in the PFA course. Similarly, 72.5% of participants in the blended course reported finding it very useful, compared to 57.4% in the MHFA eLearning group and 44.3% in the PFA group. When asked if they would use the training material in future, 89.1% of those in the eLearning group, 89.0% of those in the blended MHFA group and 73.4% of those in the PFA group reported that they would. When asked if they would recommend the course to others, 72.3% of those in the eLearning group, 83.5% of those in the blended MHFA group and 62% of those in the PFA group reported that they definitely would. Differences between groups on these last four questions all reached statistical significance at *p* < 0.05 level or lower.

## Discussion

Both the eLearning MHFA and blended MHFA courses had positive effects compared to PFA eLearning on the primary outcome of mental health first aid knowledge and the secondary outcomes of beliefs about professional treatments, quality of helping intentions and confidence in helping and personal stigma (related to dangerousness and unpredictability) towards a person with depression or PTSD. Thus, the first hypothesis was mostly supported. These findings are consistent with the growing number of studies demonstrating the effectiveness of MHFA courses [7], including two RCTs, one of which was conducted in Australian employees [12] and the other, more recently, in UK medical students [26].

In this study, the effects were similar for the eLearning and blended modes of MHFA courses, with most outcomes showing very small non-significant differences between the modes. There were some signs of blended MHFA being superior to eLearning MHFA, with significantly greater improvements in recognition of depression and quality of intentions to help a person with depression, although not for PTSD. Thus, the second hypothesis was mostly not supported.

This study makes a valuable contribution to the literature on the impact of modes of learning, which until now, has largely focused on students enrolled in formal education, who may be expected to have higher levels of motivation for attending and completing online courses [13, 14]. Poor retention rates in online studies are common and a key aim of the trial was to explore whether the addition of a face-to-face component was worthwhile in terms of learner outcomes in the MHFA course. It is notable that participants in the blended MHFA group were significantly more likely to complete the online course (64.8% vs 55.3%) and there were also trends towards a higher number of completed modules and more time spent on the course overall among participants in that group. Participants were also more likely to rate the blended MHFA course highly in terms of usefulness, amount learned and intentions to recommend to others, thus partly supporting the third hypothesis. It is therefore possible that blended MHFA may lead to improved behavioural changes in the longer term, such as a greater likelihood of participants providing more appropriate help to a person who develops a mental health problem. Data being collected at 1- and 2-year follow-up may shed more light on this, potentially adding to a sparse literature in the area of longer-term impacts of such training.

Additionally, one third of participants spent less than two hours online in total, despite being given permission to do the training in work time and the convenience of being able to log in as often as they wished and on more than one computer. Several factors may have contributed to the lower than expected usage, including a Victorian state government election, a department restructure (which led to email address changes) and the fact that the department-supplied internet browser was not optimal for the eLearning program. Such implementation difficulties are often seen in workplace-based interventions and complicate efforts to evaluate programs and to more widely disseminate those that are effective [27, 28].

Greater increases in depression recognition and helping intentions in the blended MHFA group may have arisen due to the in-depth discussion and mental health first aid skills practice that took place in the face-to-face component of the blended MHFA course. For example, in the blended MHFA course, there were three additional case studies, and a role play where participants practiced implementing the MHFA action plan and could then debrief with other participants and their instructor.

Strengths of the study include the use of a control intervention closely matched to the MHFA courses in time commitment and mode of delivery, while limitations include the larger than expected attrition and consequent lack of power to assess differences between the two modes of MHFA delivery. Furthermore, the majority of participants missing at post-test were those who did not complete their assigned course. An additional limitation is the fact that intentions may not translate into actual behaviours, although there is some evidence that mental health first aid intentions at baseline are associated with actual behaviour at follow-up [29, 30]. As part of the current study, data on actual behaviours will be collected at 1- and 2-year follow-up. Future studies could incorporate role plays using simulated situations to evaluate the impact of MHFA training on participant skills, although such methods may be better suited to training of health professionals than members of the public [31].

## Conclusions

Both blended and eLearning MHFA courses are more effective in improving knowledge, attitudes and behavioural intentions than a control intervention. However, the blended MHFA course was minimally more effective than the eLearning MHFA course in improving recognition of depression and quality of intentions to help a person with depression Course satisfaction ratings were higher in those in the blended course, potentially leading to greater benefits in the future. Longer-term follow-up is needed to explore this.

## Abbreviations

ACT: Australian Capital Territory
EAP: Employee Assistance Program
MHFA: Mental Health First Aid
OR: Odds ratio
PFA: Provide First Aid
PTSD: Post-traumatic stress disorder
RCT: Randomised controlled trial
SD: Standard deviation
VIC: Victoria
